# Structural insights into the substrate binding adaptability and specificity of human O-GlcNAcase

**DOI:** 10.1038/s41467-017-00865-1

**Published:** 2017-09-22

**Authors:** Baobin Li, Hao Li, Chia-Wei Hu, Jiaoyang Jiang

**Affiliations:** Pharmaceutical Sciences Division, School of Pharmacy, University of Wisconsin-Madison, Madison, WI 53705 USA

## Abstract

The O-linked β-*N*-acetyl glucosamine (O-GlcNAc) modification dynamically regulates the functions of numerous proteins. A single human enzyme O-linked β-*N*-acetyl glucosaminase (O-GlcNAcase or OGA) hydrolyzes this modification. To date, it remains largely unknown how OGA recognizes various substrates. Here we report the structures of OGA in complex with each of four distinct glycopeptide substrates that contain a single O-GlcNAc modification on a serine or threonine residue. Intriguingly, these glycopeptides bind in a bidirectional yet conserved conformation within the substrate-binding cleft of OGA. This study provides fundamental insights into a general principle that confers the substrate binding adaptability and specificity to OGA in O-GlcNAc regulation.

## Abstract (web summary)

O-linked β-N-acetyl glucosamine (O-GlcNAc) is an important protein modification that is hydrolyzed by O-GlcNAcase (OGA). Here the authors give insights into OGA substrate recognition by presenting four human OGA structures complexed with glycopeptide substrates containing a single O-GlcNAc modification on either a serine or threonine.

## Introduction

A diverse array of cellular processes including signal transduction and gene expression are regulated by an essential O-linked β-*N*-acetyl glucosamine (O-GlcNAc) modification of proteins, termed O-GlcNAcylation^1^. This dynamic process is controlled by the balanced activities of two opposing human enzymes: O-GlcNAc transferase (OGT) that installs O-GlcNAc on serine and threonine residues^2, 3^, and O-GlcNAcase (OGA) that specifically hydrolyzes this modification^4^. O-GlcNAcylation plays critical roles in vivo, and its dysregulation has been associated with various diseases, such as cancer, type II diabetes, and neurodegeneration^5^. Hence, there is a significant interest in understanding how OGT and OGA regulate this modification on a broad range of substrates that lack apparent sequence motifs. Structural studies revealed that the active site of OGT mainly relies on backbone interactions with various peptide substrates^6–9^. In contrast, the substrate specificity of OGA remains elusive, largely because of a long-standing challenge of crystallizing this human glycosidase.

OGA contains an N-terminal catalytic domain with sequence homology to glycoside hydrolase family 84 (GH84), a stalk domain, and a C-terminal pseudo-histone acetyltransferase (HAT) domain^10^. Previous biochemical and structural investigations on OGA bacterial homologs provided substantial insights into the mechanism of O-GlcNAc hydrolysis in the catalytic site^11–19^. However, the sequences of OGA’s stalk domain and HAT domain bear significant variations from the bacterial homologs, therefore, how OGA recognizes diverse substrates beyond the catalytic pocket remains elusive. Recently, our group and others have independently identified crystallizable constructs of OGA that comprise the catalytic domain and stalk domain, and published the apo form structures and enzyme complexes with active site inhibitors^20–22^. These reports consistently showed that OGA formed an unusual arm-in-arm homodimer, where the catalytic domain of one monomer covered by the stalk domain of the sister monomer to create a potential substrate-binding cleft. We further determined the structure of OGA in complex with a p53 glycopeptide and provided a direct view into the substrate-binding state of this glycosidase^20^. Notably, we found that the p53 glycopeptide was tightly bound in the substrate-binding cleft through abundant contacts of GlcNAc in the OGA catalytic pocket, and via peptide side chain and backbone interactions with cleft surface residues. These observations suggest that besides the GlcNAc moiety, OGA enables recognition of specific features of substrate peptides. It has been reported that OGA can hydrolyze O-GlcNAc from a broad range of peptide sequences^16^, but it is unclear whether OGA binds all glycopeptides in the same orientation or conformation. In the present study, we aim to assess the generality of the substrate-binding mode of OGA and to extend our understanding on the principle of OGA substrate recognition.

## Results

### Structures of OGA_cryst_-D175N and its glycopeptide complexes

Exploiting our previously reported construct of OGA_cryst_ (residues 60–704 with the unstructured insert residues 401–552 replaced by a glycine–serine (GS) linker)^20^, we solved the structures of a mutant OGA_cryst_-D175N (catalytically impaired but retaining the ability to bind substrate) in apo form and in complex with each of four synthetic glycopeptides. These glycopeptides were derived from characterized O-GlcNAcylation sites in the proteins: (a) α-crystallin B chain (FPT**S**TSLSPFYLR);^9^ (b) TAB1 (VPY**S**SAQS);^16^ (c) ELK1 (FWS**T**LSPI);^9^ and (d) Lamin B1 (KLSPSPSSRV**T**VS)^9^ (Table 1). Each of these peptides contains a single O-GlcNAc modification on the highlighted serine or threonine that is flanked by distinct amino acids. Even though the peptide terminal residues lacked electron density, indicating that they could adopt a variety of binding conformations, most residues adjacent to the O-GlcNAcylation sites showed clear density and were refined with occupancies of 1.0. Of note, the complexes of ELK1 and Lamin B1 represent OGA substrate structures with an O-GlcNAcylated threonine.

**Table 1:** Data collection and refinement statistics (molecular replacement)

	OGA_cryst_-D175N (PDB 5VVO)	OGA_cryst_-D175N–α crystallin B (PDB 5VVV)	OGA_cryst_-D175N–TAB1 (PDB 5VVU)	OGA_cryst_-D175N–ELK1 (PDB 5VVT)	OGA_cryst_-D175N–Lamin B1 (PDB 5VVX)
*Data collection*					
Space group	*P*21	*P*21	*P*21	*P*21	*P*21
Cell dimensions					
* a*, *b*, *c* (Å)	82.4, 96.8, 89.1	83.1, 96.3, 89.8	82.5, 96.1, 88.9	82.4, 95.8, 89.3	82.9, 96.2, 89.7
* α*, *β*, *γ* (°)	90.0, 115.2, 90.0	90.0, 114.3, 90.0	90.0, 114.5, 90.0	90.0, 114.5, 90.0	90.0, 114.6, 90.0
Resolution (Å)	50.0–2.6 (2.64–2.60)^a^	50.0–2.7 (2.80–2.70)	50.0–2.7 (2.75–2.70)	50.0–2.8 (2.85–2.80)	50.0–2.9 (2.95–2.90)
*R*_merge_	8.4 (63.1)	7.1 (93.5)	8.0 (68.0)	8.4 (72.2)	8.9 (69.0)
*I*/σ*I*	21.9 (2.0)	18.8 (1.5)	22 (1.6)	17.8 (1.8)	20.4 (1.8)
*CC*_1/2_	99.9 (83.3)	98.6 (58.7)	99.0 (70.7)	99.3 (73.1)	99.7 (74.5)
Completeness (%)	99.3 (93.3)	99.8 (98.6)	99.8 (97.3)	99.9 (99.7)	99.8 (98.9)
Redundancy	6.3 (5.7)	5.6 (5.5)	4.4 (3.5)	4.2 (4.1)	4.6 (4.5)
*Refinement*					
Resolution (Å)	50.0–2.6	50.0–2.8	50.0–2.7	50.0–2.8	50.0–2.9
No. reflections	38,896	31,917	34,865	31,269	28,990
* R*_work_ /*R*_free_	21.0/26.0	19.6/25.6	20.7/27.5	21.1/29.1	20.0/28.3
No. atoms					
Protein	7141	6905	7000	6920	6994
Ligand/peptide	0	75	90	71	96
Water	133	35	50	49	13
* B*-factors	60.27	74.54	68.32	70.93	90.85
Protein	60.51	74.45	68.05	70.89	90.41
Ligand/peptide		85.10	88.49	81.79	125.59
Water	47.62	58.03	62.55	56.64	68.86
R.m.s. deviations					
Bond lengths (Å)	0.012	0.013	0.012	0.012	0.012
Bond angles (°)	1.622	1.706	1.592	1.636	1.536

Each structure was determined from one crystal

^a^Values in parentheses are for highest-resolution shell

### Peptides bind in a bidirectional yet conserved conformation

**Fig. 1:**
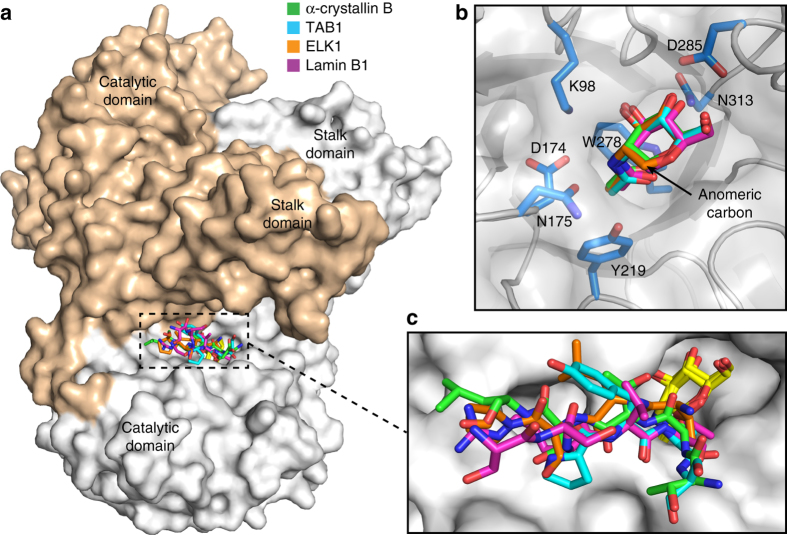
Different glycopeptides are bound in the substrate-binding cleft of OGA in a similar conformation. **a** The structure of dimeric OGA_cryst_-D175N in complex with glycopeptide substrates derived from the following proteins: α-crystallin B chain, TAB1, ELK1, and Lamin B1. The two monomers of OGA_cryst_-D175N are shown in surface representation with *white* and *wheat* color, respectively. The glycopeptides are displayed in *sticks* with indicated colors. **b** A close-up view of GlcNAc residues from different glycopeptides in the complex structures. The coloring of GlcNAc from each glycopeptide is indicated in **a**. The same set of OGA residues participating in the interactions with GlcNAc are shown in *marine blue sticks* and labeled with residue numbers. **c** Enlarged view (*boxed area*) of the active site region of OGA (*gray surface*) demonstrates that different glycopeptide substrates are bound in a similar conformation. The GlcNAc residues are shown in *yellow sticks*. The sister monomer of OGA has been removed and the glycopeptides have been rotated for better clarity

A structural overlay of OGA_cryst_-D175N in the substrate-free and substrate-bound states illustrated that substrate binding did not induce any changes in the dimeric structure of OGA (Supplementary Fig. 1). In the substrate complexes, all the glycopeptides were bound in the OGA substrate-binding cleft (Fig. 1a). Particularly, the GlcNAc moieties were anchored by the same set of OGA residues in the catalytic pocket, displaying nearly identical binding conformations (Fig. 1b) regardless of the glycosylated residues or the flanking peptide sequences. However, when superimposing these glycopeptides with the reported p53 glycopeptide in the OGAα monomer (PDB: 5UN8) that is free of crystal packing impact^20^, the α-crystallin B chain and ELK1 peptides were orientated opposite to that of TAB1, Lamin B1, and p53 (Supplementary Fig. 2a). Strikingly, we found that these peptide backbones adopted a similar binding conformation, with the four peptide backbones exhibiting a more elongated conformation than the curved p53 (Fig. 1c and Supplementary Fig. 2a). This slight conformational deviance was likely a result of the four peptide backbones lacking the strong intra-molecular hydrophobic interactions of p53 that contorted the peptide termini (Supplementary Fig. 2b). These structures indicate that OGA is capable of binding peptide substrates in a bidirectional yet generally conserved conformation.

### Comparison of OGA interactions with distinct glycopeptides

**Fig. 2:**
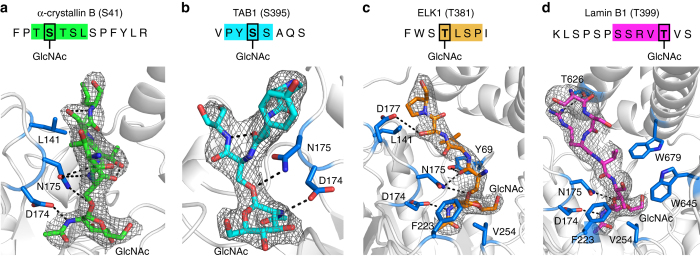
Comparison of OGA interactions with distinct glycopeptides. The sequences, conformations, and representative 2*Fo*–*Fc* electron density maps (*gray*) of four glycopeptides bound in the substrate-binding cleft of OGA, contoured at 1.0 σ. O-GlcNAcylated peptides: **a** α-crystallin B chain, **b** TAB1, **c** ELK1, and **d** Lamin B1. On the *top* of each panel, the glycopeptide sequence is displayed. The peptide residues observed in the crystal structure are highlighted with *colored background* and the O-GlcNAcylation site is highlighted by a *black box*. At the *bottom* of each panel, the binding conformation of each peptide is shown in *sticks* with the same color as its highlighted sequence. The residues of OGA participating in the interactions with each peptide are shown in *marine blue sticks* and labeled with residue numbers. Hydrogen bonds are displayed as *dashed lines*

We further examined the interactions of each peptide to gain molecular insights into the principle of OGA substrate recognition. Consistent with the O-GlcNAc hydrolysis mechanism^13, 20^, the catalytic residue D174 was optimally positioned to make a hydrogen bond with the *N*-acetyl group of GlcNAc in all the complexed structures (Fig. 2). The N175 mutant side chain played an important role in anchoring the glycosylated hydroxyl of serine or threonine and stabilizing the OGA substrate complexes. Compared to the glycosylated serine, the extra methyl group of the glycosylated threonine (as found in ELK1 and Lamin B1 peptides in Fig. 2c, d) was well accommodated in the hydrophobic pocket harboring F223 and V254 residues from the top surface of the catalytic domain and W679 from the stalk domain of the sister monomer. In addition to these common features, peptide specific interactions have also been detected. As an example, the α-crystallin B chain was stabilized in a V shape by a pair of hydrogen bonds between the peptide backbone and the N175 residue (Fig. 2a). The hydroxyl groups of threonine (+ 1 subsite) and serine (+ 2 subsite) were further stabilized by intra-molecular hydrogen bonds, while the aliphatic side chain of leucine (+ 3 subsite) made favorable van der Waals contacts with L141 on the inner surface of the substrate-binding cleft (Fig. 2a). Intriguingly, the peptide backbone of TAB1 also adopted a V shape, but it was stabilized by an intra-molecular hydrogen bond instead of the interactions with N175 (Fig. 2b). In the structure of OGA-ELK1 complex (Fig. 2c), residues Y69 and N175 anchored the peptide backbone. Furthermore, side chain specific interactions with cleft surface residues reinforced the binding (Fig. 2c). Finally, in the OGA-Lamin B1 complex, the peptide was primarily stabilized by side chain specific interactions with the cleft surface residues (Fig. 2d): valine (-1 subsite) was engaged in hydrophobic and van der Waals interactions with W645, whereas arginine (-2 subsite) participated in forming a hydrogen bond with T626. These findings support that the substrate-binding cleft affords distinct interactions to coordinate a variety of peptide sequences, endowing OGA with adaptability and specificity for substrate binding during the dynamic O-GlcNAc regulation.

## Discussion

OGA is the unique enzyme responsible for O-GlcNAc hydrolysis from a large number of cytoplasmic and nuclear proteins. Emerging evidence showed that O-GlcNAcylation turnover rate varied substantially on different proteins^23^, suggesting that OGA quickly removes O-GlcNAc from certain substrates while leaving unfavorable substrates more stably modified. Hence, there is a great interest in understanding how OGA recognizes various substrates and dynamically regulates O-GlcNAc biology. Towards addressing this important question, bacterial OGA homologs have been crystallized^13, 14, 18^ and two were solved in complex with synthetic glycopeptides^16, 17^. These studies illustrated how the GlcNAc moiety is anchored by a set of highly conserved residues in OGA catalytic pocket. However, the peptide-binding conformations from these studies could not be directly applied to human OGA. A major difference between human OGA and its bacterial homologs is that human OGA is a dimeric protein featured with a substrate-binding cleft. Exploiting a recently identified crystallizable construct of human OGA (OGA_cryst_)^20^, we determined its structures in complex with different glycopeptides. Intriguingly, we found that the glycopeptides bound in the substrate-binding cleft in a bidirectional yet nearly identical conformation. In addition, we noted that the same TAB1 glycopeptide was previously reported as V shaped in bacterial OGA complexes: *Cp*OGA-TAB1 (PDB: 2YDS)^16^ and *Tt*OGA-TAB1 (PDB: 5DIY)^17^. However, the peptide residues of TAB1 oriented dramatically differently in human OGA from those in the bacterial homologs (Supplementary Fig. 3), suggesting that human OGA dimer employs a unique substrate-binding mode.

From this and our previous studies on the structures of total five OGA-peptide complexes, a general principle for OGA recognizing various peptide substrates can be deduced. The abundant and conserved interactions between the GlcNAc moiety and OGA catalytic site secure the binding of the glycopeptide within the substrate-binding cleft. These interactions serve as a prevalent driving force for OGA to selectively target O-GlcNAcylated substrates in the whole proteome. Enhanced selectivity can be achieved through substrate-specific interactions between OGA cleft surface residues and the substrate peptides. If the peptide bears intra-molecular interactions, it would provide additional stabilization energy for maintaining its ordered binding conformation. Based on the features of peptide binding outside the catalytic pocket of OGA, we propose that OGA is able to recognize and discriminate its substrates.

In summary, we report the structures of OGA in complex with different glycopeptide substrates. We find that OGA is able to bind peptide substrates in different directions, but in a conserved conformation regardless of the glycosylation site or flanking sequences. Notably, the OGA substrate-binding cleft affords distinct interactions to coordinate a variety of peptide sequences, providing critical insights into a general principle that confers the substrate binding adaptability and specificity to OGA. The knowledge obtained from this study will substantially advance understanding on the regulatory role of OGA in O-GlcNAc biology, and will facilitate rational design of substrate-specific inhibitors to block OGA dysfunction for biomedical use.

## Methods

### Protein expression and purification

The OGA mutant (referred as OGA_cryst_-D175N comprising 60–704 residues of human OGA with the unstructured 401–552 region replaced by a glycine–serine linker) was prepared similarly as previously described^20^. Briefly, the DNA encoding mutant OGA-D175N was cloned into a modified pET-SUMO vector (primers are listed in Supplementary Table 1) and transformed into *Escherichia coli* strain Rosetta (DE3) (Novagen) for protein expression. The cells were harvested, resuspended, and homogenized with an ultra-high-pressure cell disrupter (Emulsiflex-C5, Canada). The supernatant was purified by Ni–NTA resin (Qiagen) at 4 °C and the desired protein was eluted by buffer containing 20 mM Tris (pH 8.0), 150 mM NaCl, and 250 mM imidazole. The eluted protein was digested by Sumo protease to remove the 6 × His–SUMO tag and further purified by size-exclusion chromatography (Superdex 200 increase 10/300, GE Healthcare) using buffer containing 20 mM Tris (pH 8.0), 150 mM NaCl, and 0.5 mM THP (Tris(hydroxymethyl)phosphine, EMD). The OGA_cryst_-D175N protein was concentrated to 3 mg ml^−1^ for crystallization.

### Crystallization

All of the crystals were generated by mixing 1 μl of protein with an equal volume of reservoir solution and were equilibrated against 200 μl of reservoir solution using the hanging-drop vapor-diffusion method at 20 ^o^C. Native OGA_cryst_-D175N crystals were obtained in the reservoir solution containing 0.032 M ammonium citrate tribasic (pH 7.0), 0.02 M MES monohydrate, 0.128 M potassium thiocyanate, 0.016 M imidazole, 0.002 M zinc sulfate heptahydrate, 12.8% w/v polyethylene glycol 3350, 3.2% w/v polyethylene glycol monomethyl ether 2000, and 5% w/v polyethylene glycol monomethyl ether 550. Glycopeptide complexes were obtained through soaking the native crystals in reservoir solution containing 5–10 mM of each glycopeptide (prepared by solid-phase peptide synthesis) for 1–2 h prior to cryoprotection with 10% glycerol in mother liquor. The crystals were flash-frozen in liquid nitrogen for storage.

### Data collection and structure determination

All the X-ray data were collected on the Life Sciences Collaborative Access Team (LS-CAT) beam lines 21-ID-G (for OGA_cryst_-D175N apo form, OGA_cryst_-D175N–α-crystallin and OGA_cryst_-D175N–TAB1 complexes) and 21-ID-F (for OGA_cryst_-D175N–ELK1 and OGA_cryst_-D175N–Lamin B1 complexes) (LS-CAT, Advanced Photon Source, Argonne National Laboratory, IL, USA). The wavelength for data collection was 0.9787 Å. All data sets were processed using the HKL2000 package^24^. The crystals of glycopeptide complexes all belonged to the *P*2_1_ space group, with two molecules per asymmetric unit. The structures were solved by molecular replacement using OGA_cryst_ as a search model (PDB: 5TKE)^20^. Iterative model building was performed in COOT^25^, followed by refinement with PHENIX^26^ and Refmac5^27^. Final refinement statistics were summarized in Table 1. Structural figures were prepared using the program PyMOL^28^.

### Data availability

Coordinates and structural factors have been deposited in the Protein Data Bank under accession codes 5VVO, 5VVV, 5VVU, 5VVT, and 5VVX for OGA_cryst_-D175N, OGA_cryst_-D175N–α-crystallin, OGA_cryst_-D175N–TAB1, OGA_cryst_-D175N–ELK1, and OGA_cryst_-D175N–Lamin B1, respectively. All other data are available from the corresponding author upon reasonable request.

## Electronic supplementary material

Supplementary Information
