# Supplementary material for: Structural insights into the substrate binding adaptability and specificity of human O-GlcNAcase
Source: Nat Commun. 2017 Sep 22;8:666. doi: 10.1038/s41467-017-00865-1 (PMC5610315; doi:10.1038/s41467-017-00865-1)
Supplement: Supplementary file 1 — Supplementary Information [file 41467_2017_865_MOESM1_ESM.pdf]

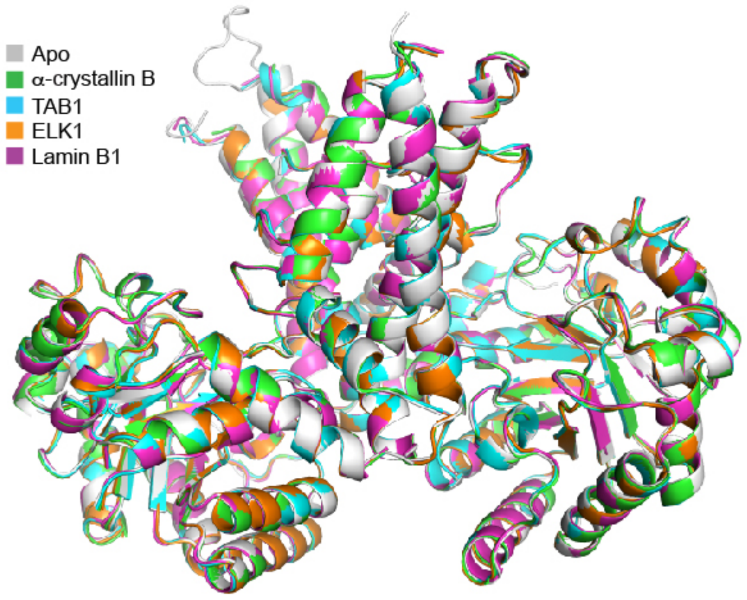

Supplementary Figure 1. Superposition of the apo form structure of OGA_{cryst}-D175N with its glycopeptide complexes shows that substrate binding did not induce any changes in the OGA dimeric structure. The OGA_{cryst}-D175N proteins in apo form structure and complexes with α-crystallin B chain, TAB1, ELK1 and Lamin B1 glycopeptides are shown in ribbon representation and colored by grey, green, cyan, orange and magenta, respectively.

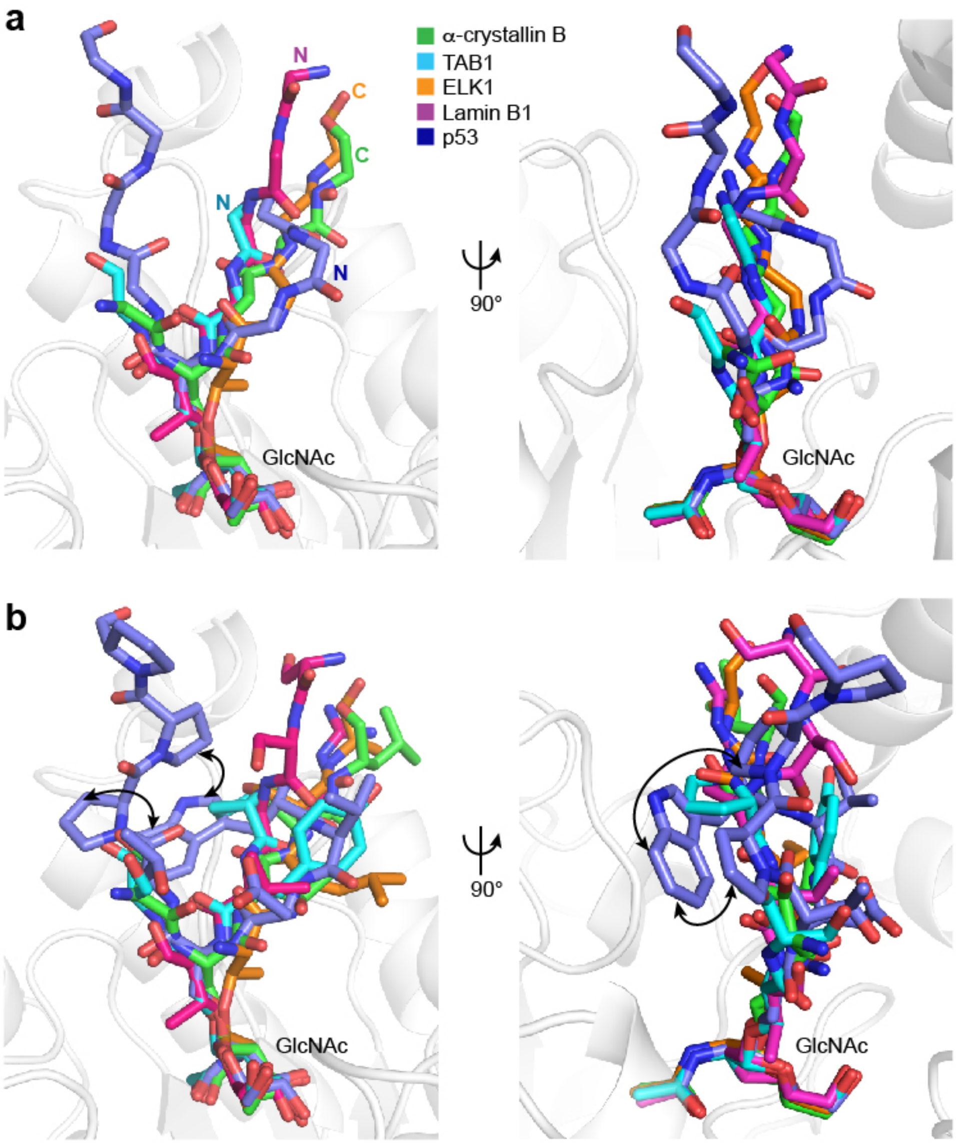

Supplementary Figure 2. Compared to the reported p53 glycopeptide, the four glycopeptides in this study are bound in an analogous but more extended conformation in the substrate-binding cleft of OGA. (**a**) Two different views of the backbone superposition of five glycopeptides in the complex structures of OGA_{cryst}-D175N illustrate that they are bound in a generally similar conformation with the backbone of p53 glycopeptide slightly more bent. The backbones of glycopeptides α-crystallin B chain, TAB1, ELK1, Lamin B1, and p53 (PDB: 5UN8)[1] are shown in green, cyan, orange, magenta and blue sticks, respectively. The peptide side chains have been hidden for clarity. In the left panel, the N- or C-terminus of each peptide is labeled in order to show that OGA binds glycopeptides in bidirectional orientations. (**b**) Two different views of the five glycopeptides in the complex structures of OGA_{cryst}-D175N, in which the peptides are shown in sticks. The intra-molecular hydrophobic interactions of p53 glycopeptide (PDB: 5UN8) are indicated by black arrows and could be attributed to the more bent conformation of p53 when compared to other OGA bound glycopeptides.

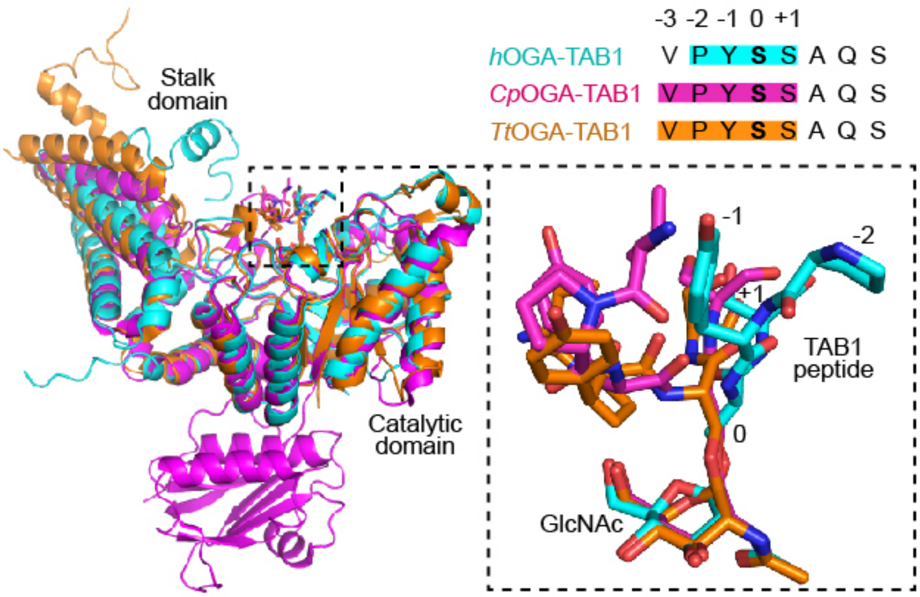

Supplementary Figure 3. TAB1 glycopeptide adopts distinct binding conformations in the complex structures of human and bacterial OGAs. Comparison of the binding conformations of TAB1 glycopeptides in the structures of human OGA_{cryst}-D175N–TAB1, *Cp*OGA–TAB1 (PDB: 2YDS)[2], and *Tt*OGA–TAB1 (PDB: 5DIY)[3]. Human OGA, *Cp*OGA and *Tt*OGA are shown in ribbon with cyan, magenta and orange color, respectively. The TAB1 glycopeptides are indicated in sticks, the peptide residues observed in the crystal structures are highlighted by indicated colors, and the O-GlcNAcylated serine residues are highlighted in bold.

Supplementary Table 1. Summary of primers used to make OGA mutant in this study.

Primer	Sequence (5′-3′)
D175N_fwd	GATCATTTGCTTTGCTTTTTGATAATATAGACCATAATATGTGTGCAGC
D175N_rev	CACATATTATGGTCTATATTATCAAAAAGCAAAGCAAATGATCTGCAC

Supplementary References

1. Li, B., Li, H., Lu, L. & Jiang, J. Structures of human O-GlcNAcase and its complexes reveal a new substrate recognition mode. *Nat. Struct. Mol. Biol.* **24,** 362–369 (2017).
2. Schimpl, M., Borodkin, V. S., Gray, L. J. & van Aalten, D. M. F. Synergy of peptide and sugar in O-GlcNAcase substrate recognition. *Chem. Biol.* **19,** 173–178 (2012).
3. Ostrowski, A., Gundogdu, M., Ferenbach, A. T., Lebedev, A. A. & van Aalten, D. M. F. Evidence for a functional O-linked N-acetylglucosamine (O-GlcNAc) system in the Thermophilic bacterium *Thermobaculum terrenum*. *J. Biol. Chem.* **290,** 30291–30305 (2015).
